# Hurler Syndrome: Orofacial Clinical Findings

**DOI:** 10.7759/cureus.33313

**Published:** 2023-01-03

**Authors:** Cristina Rodrigues Barros, José Ferrão, Maria do Céu Machado, Ana Fernandes, Francisco Proença

**Affiliations:** 1 Stomatology Department, Centro Hospitalar Universitário de Lisboa Central, Lisbon, PRT; 2 Pediatric Stomatology Department, Centro Hospitalar Universitário de Lisboa Central, Lisbon, PRT

**Keywords:** oral & maxillofacial pathology, pediatrics, hurler syndrome, mucopolysaccharidosis, metabolic disorder

## Abstract

Hurler syndrome (HS) belongs to the category of mucopolysaccharidosis (MPS), a spectrum of rare genetic disorders of the mucopolysaccharides metabolism. This syndrome is due to a defect in α-iduronidase, an enzyme responsible for the degradation of the glycosaminoglycans (GAGs) heparin and dermatan sulfate. Intra and extracellular accumulation of these non-metabolized substances may lead to multisystemic dysfunction, with severe stomatognathic involvement that may often need treatment. The aim of this article is to present the heterogeneity of orofacial and radiographic findings observed in two patients with HS with long-term follow-up, who were referred to our Stomatology department.

## Introduction

MPS is a rare (4:100,000 newborns) and heterogenous group of metabolism genetic disorders [1-4]. Characterized by a deficiency of the enzymes responsible for the degradation of glycosaminoglycans (GAGs), it can lead to multisystemic morbidity and often dictates the need for multidisciplinary management [5]. To date, seven different types of MPS are described (I, II, III, IV, VI, VII, IX), determined by which of the 11 enzyme defects identified are found [6].

Mucopolysaccharidosis I-Hurler (MPS I-H) was clinically described in 1919, by Gertrud Hurler, a German pediatrician [7-8]. It is the most severe form of MPS caused by mutation of the alpha-iduronidase gene, which encodes the alpha-L-iduronidase enzyme [7]. MPS I-H is a life-threatening disease, involving progressive neurological disease, mental retardation, skeletal malformations, upper airway obstruction, and cardiac, ophthalmological, and orofacial changes [9-10]. Among the clinical features, that involve the head and neck regions, are gargoyle-like facies with hypertelorism, a prominent forehead and supraorbital ridges, scaphocephaly, flattening of the nasal bridge with a snub nose and large nostrils, and short neck [11]. The range of intraoral abnormalities is vast and can be observed either clinically or radiographically. Broadly comprehends dental anomalies (spaced hypoplastic peg-shaped teeth), delayed eruption, increased risk of dental caries/periodontal disease (hyperplastic gingiva), macroglossia, maxillary and mandibular changes (high-arched palate, short mandibular rami with abnormal condyles, localized dentigerous cyst-like radiolucency), thick lips, malocclusion, and temporomandibular joint disorder [1,5,12-16].

## Case presentation

Case 1

The first case describes a female patient, first observed at seven years old, referred by the department of Metabolic Diseases to the Stomatology department for a routine dental examination. The initial diagnosis of HS was made at four years old, and since then, she has been receiving treatment with weekly enzyme replacement therapy.

**Figure 1:**
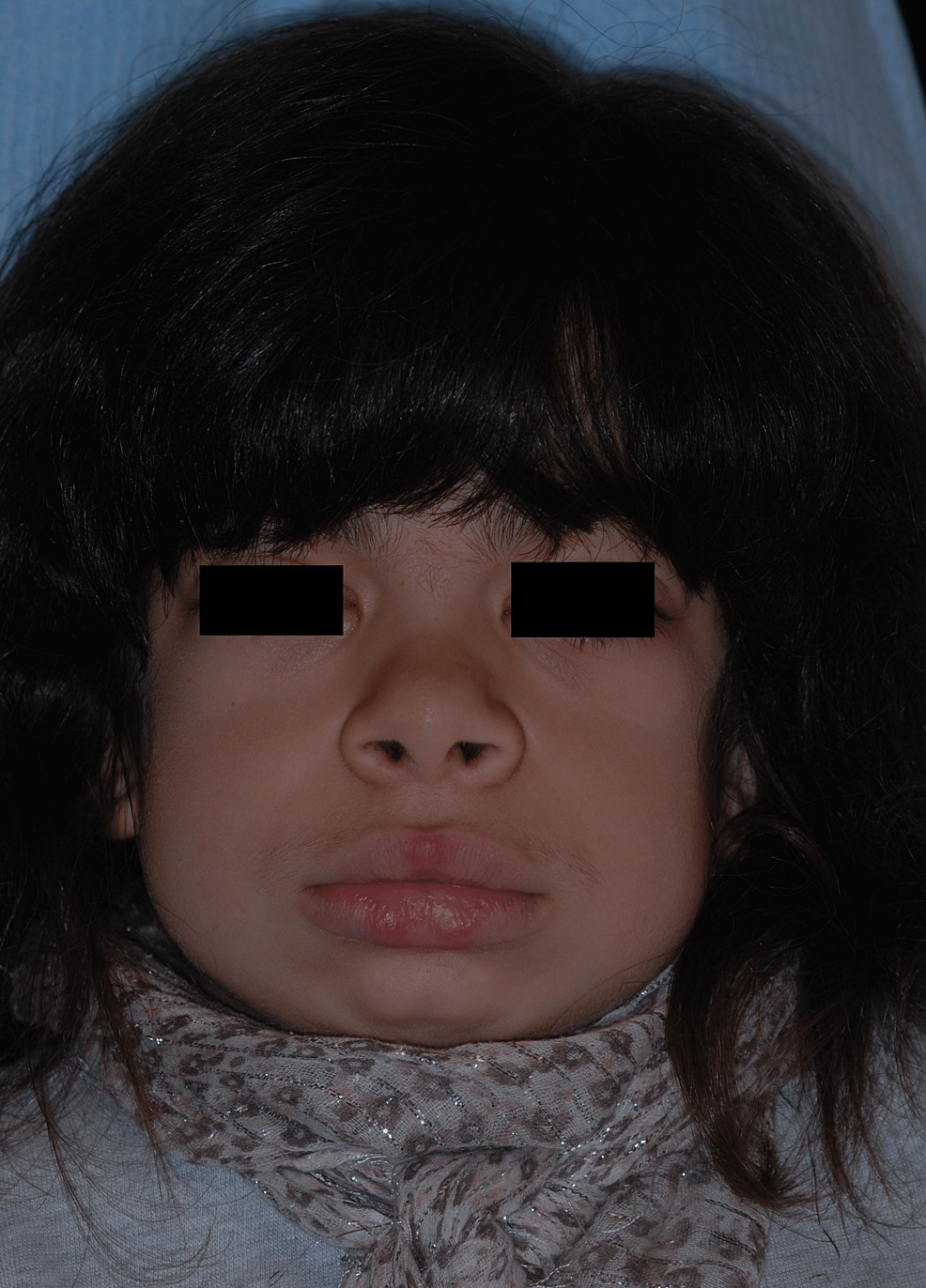
Frontal facial view showing coarse features: large head, short neck, broad nose tip, and thickened lips with flattened philtrum

Clinically, the patient had an intellectual disability, bilateral corneal opacity, adenotonsillar hypertrophy, mitral insufficiency, carpal tunnel syndrome, growth retardation, and bone deformities, specifically, atlantoaxial instability and acetabular dysplasia. On examination, the following facial features were observed: hypertelorism, flat nasal bridge, flared nose, upturned nasal tip, large mouth with broad lips, and a discrete anterior open bite (Figure 1).

**Figure 2:**
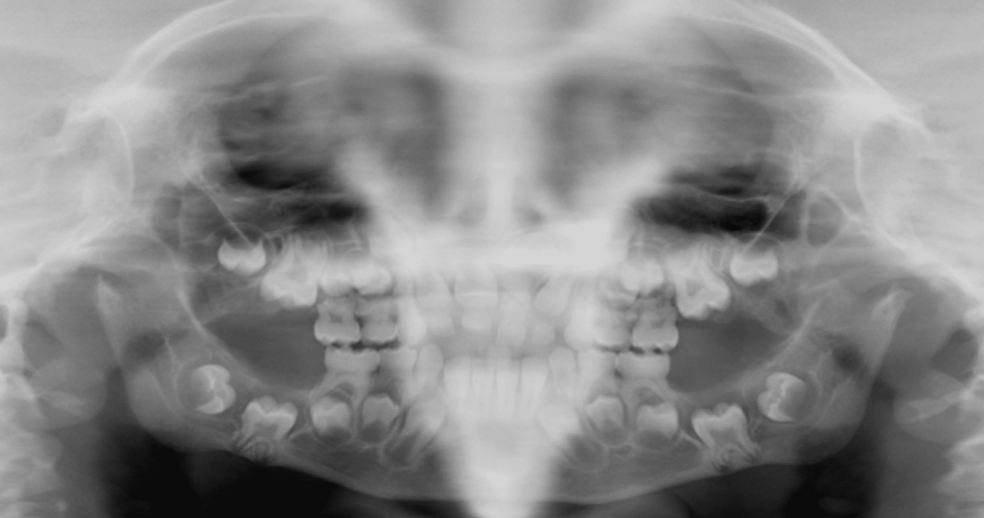
Orthopantomography: cystic lesions with erupting impacted permanent teeth and bilateral hypoplasia of mandibular condyles with shortened vertical ramus height.

Comprehensive intraoral examination was constrained by poor cooperation and limited mouth opening. Fortuitously, compliance allowed an orthopantomography. The image revealed a dental age delay with incompleted rhizogenesis of all first molars, a maxillary permanent central incisive (21) impacted by one supernumerary tooth, and four supernumerary molar teeth. An irregular jaw arch was also seen with cyst-like areas of bone destruction surrounding unerupted permanent molar teeth and ramus of the mandible (Figure 2).

The hypoplastic appearance of the mandibular condyles and dysmorphic glenoid cavities confirmed the clinical finding of anterior open-bite and limited opening of the mandible.

At eight years old, she was surgically managed under general anesthesia. The anesthetic procedure was complicated by airway obstruction, but the surgery was successfully performed. A mucoperiosteal flap was lifted with the extraction of the supernumerary tooth with plasty of the upper labial frenum.

**Figure 3:**
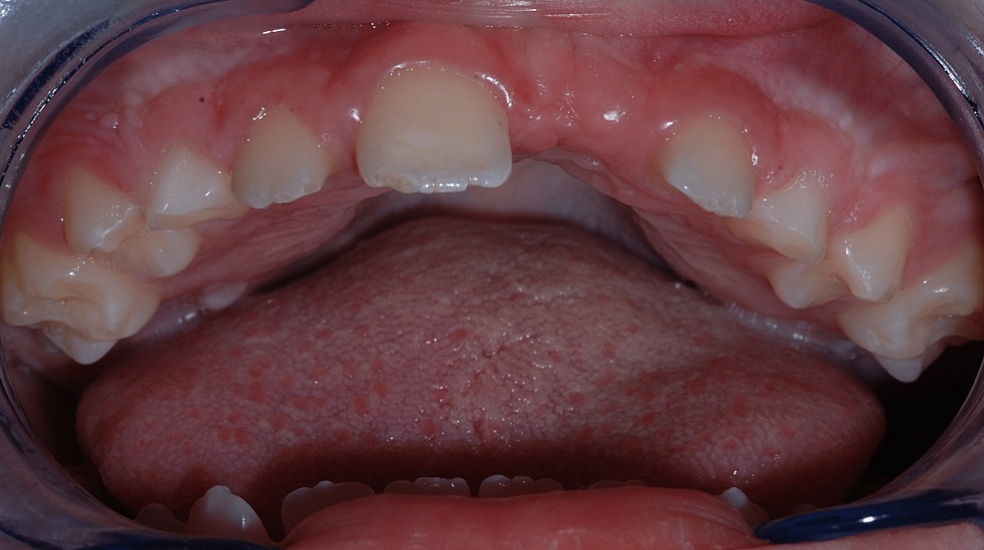
Upper arch: unerupted tooth 21

At the two-year follow-up consultation, a clinical intraoral evaluation revealed ogival palate, macroglossia, absence of tooth 21 in the arcade that was palpated in the vestibular mucosa, and non-carious lesions on primary and permanent teeth that were widely spaced with gingival tissues notably thickened (Figure 3).

**Figure 4:**
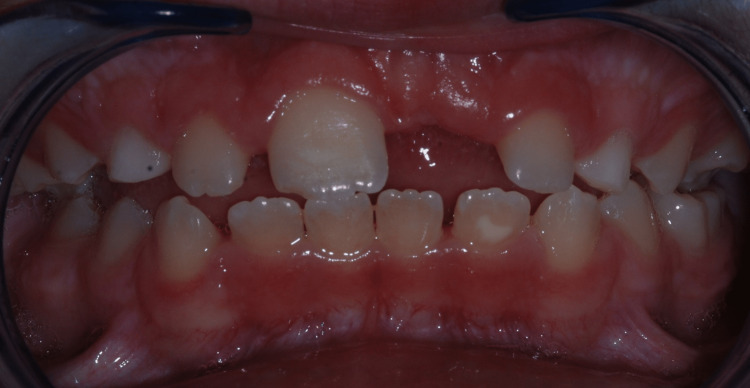
Intraoral photograph: spacing between permanent teeth and enamel pitting

The enamel of primary and permanent teeth was slightly hypoplastic with pitted enamel (Figure 4).

Four years later, the patient remains on regular follow-up, without new complaints and intraoral dental or mucosal lesions, to ensure maintenance of oral health care and reevaluation of her delayed eruption.

Case 2

**Figure 5:**
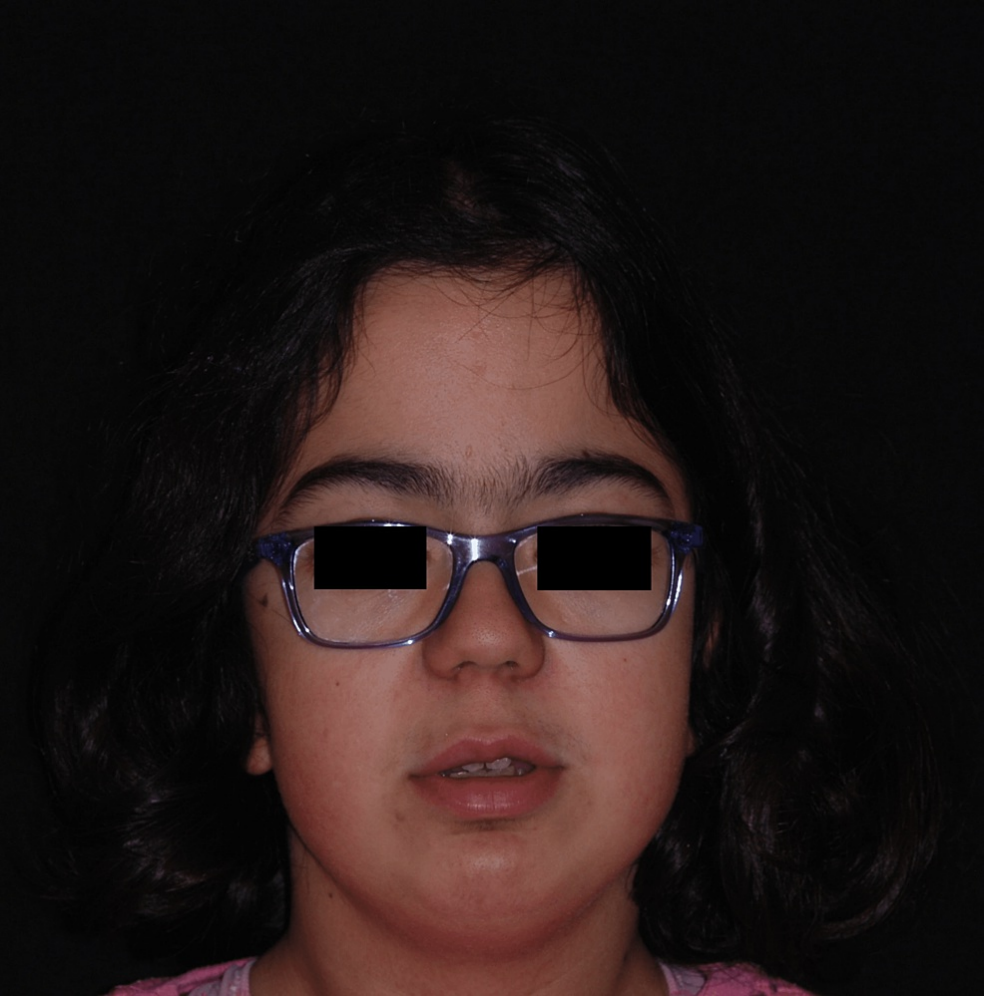
Frontal facial view

The second case describes a 12-year-old female referred at the age of seven by the department of Metabolic Diseases to the Stomatology department. A comprehensive medical history revealed that she was diagnosed with HS and had received a successful bone marrow transplant at age two with posterior chemotherapy with busulfan. Medical comorbidities included aortic regurgitation, mitral dysplasia, and reduced visual acuity with corneal opacification and osseous malformations (genu-valgum surgically corrected). Clinically, no coarse facial features were observed (Figure 5).

**Figure 6:**
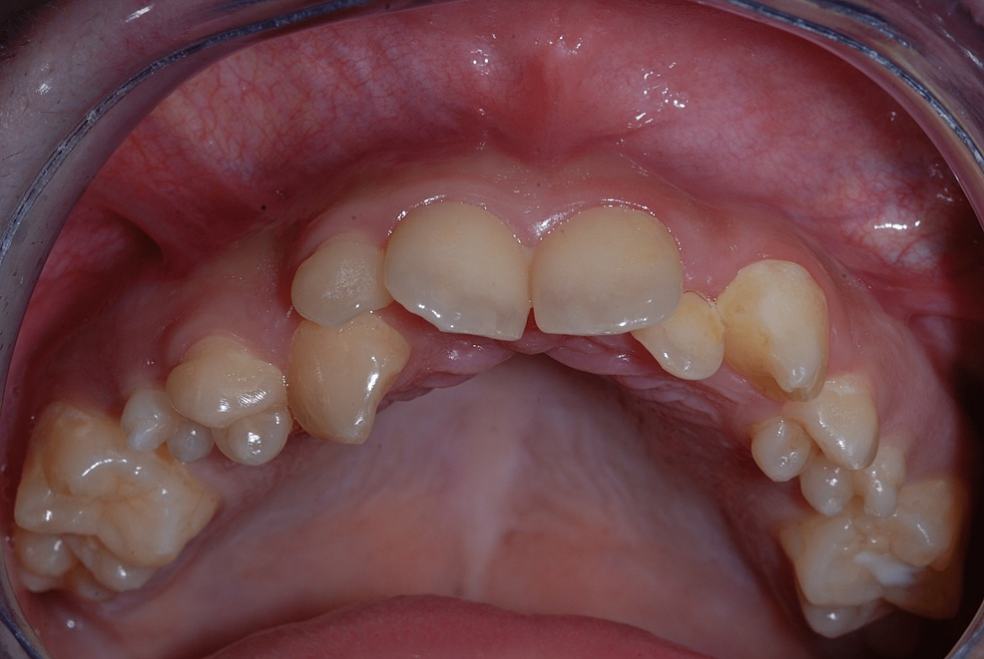
Intraoral photograph: enamel hypoplasia and dysmorphic superior premolars

**Figure 7:**
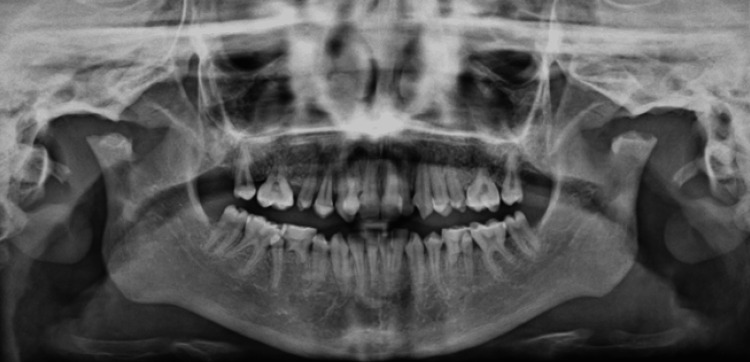
Orthopantomography: short mandible and flat irregular condyles surface

On intraoral examination, despite the presence of dental plaque, the soft tissues were healthy and no carious lesions were present. Dental and osseous abnormalities were the principal findings on physical and radiographic examination, with enamel hypoplasia of permanent teeth, dysmorphic superior premolars (14, 15, 24, and 25), and severe anatomical alteration of the condyles (Figure 6 and Figure 7).

The patient was maintained with regular follow-up, with scaling under prophylaxis to prevent endocarditis.

## Discussion

Orofacial features are dependent on the MPS subtype (Table 1) [17].

**Table 1:** Orofacial findings in MPS patients MPS: mucopolysaccharidosis

MPS subtype	Dental features	Periodontal features
MPS I	Delayed tooth eruption, malocclusion, taurodontism, hypodontia, microdontia	Gingival hyperplasia, radiolucency in maxilla / mandible, condylar defects
MPS II	Delayed tooth eruption, malocclusion	Gingival hyperplasia, radiolucency in maxilla / mandible, condylar defects
MPS III	Obliteration of pulp chambers, irregular pulp morphology	
MPS IV	Enamel defects (discoloration, hypoplasia), tooth surface loss, spaced dentition, peg-shaped incisors, cross bites	Flattened condyles
MPS IV	Tooth impaction, root resorption, taurodontism	Fibrous gingival dysplasia, dentigerous cysts, bony rarefactions, enlarged marrow spaces, osteosclerosis, TMJ dysfunction

Particularly in MPS I-H, patients have often been described with peg-shaped teeth, poorly formed, with hypoplastic enamel, like in the two cases mentioned above, where the permanent incisors were extremely pointed. Moreover, an anterior open bite is also seen, as in the first case, caused by macroglossia due to the deposition of GAGs within the tongue structure. Radiographic findings like short ramus, abnormal morphology of condyles, alteration of the articular eminence, and cystic-like lesions (mostly in the mandible) are classical in HS too, and are corroborated by the two presented cases [18].

Besides the type of MPS, the treatment modalities can likewise cause dental modification and mucosal and facial bone involvement. Treatment for MPS I-H comprises conservative management, enzyme replacement therapy, and hematopoietic stem cell transplantation (HSCT) [17].

HSCT is considered successful management of MPS I-H and has also shown improved clinical outcomes in other subtypes. While the effects of the presenting condition may be attenuated, or even eliminated, by treatment, such children will inevitably experience side effects. Changes in dental development may occur due to potential collateral effects of chemotherapy and radiotherapy, like agenesia and hypoplasia of the teeth. The second patient had a successful HSCT at 24 months of age. The odontogenesis inhibition, as a side effect of the preparatory chemotherapy, may explain hypoplasia and abnormal structure of the permanent pre-molars in this patient. This draws attention to the need for a multidisciplinary approach to discuss oral health problems related to chemotherapy on orofacial abnormalities like teeth development at the time of treatment [17-18].

Alternatively, intravenous enzyme replacement therapy may be a valuable treatment in many subtypes of MPS (namely, I, II, IV, VI). However, its use is limited to subtypes with widespread neurological symptoms since it can cross the blood brain barrier. Subtypes with neurological involvement are an additional challenge for clinicians due to profound neurological symptoms and in some cases, intellectual disability. These clinical aspects are often associated with poor cooperation for oral hygiene, and, consequently, gross caries, which are not suitable for conservative treatment and may also lead to dental intervention under general anesthesia, as seen in the first reported case [17,19]. Therefore, when an anesthesiologist is required, they must be aware of cardiac comorbidity, obstructive airway disease, and atlantoaxial instability, which are frequent in these patients and lead to elevated anesthetic risk [20].

This shows the importance of regular oral care appointments, centered on prevention with oral health promotion protocols (liaison with their metabolic dietician), intensive oral hygiene instruction, early identification of dental caries with the application of premature fissure sealants to the first permanent molars on eruption, and fluoride supplementation [17-18].

Currently, less than 30 clinical cases and no guidelines for stomatological management in these patients have been published. This fact shows the importance and need for an international consensus and the establishment of standardized guidelines for MPS management.

## Conclusions

The evidence available on orofacial manifestations and management in HS patients is scarce, making it difficult to establish preventive measures with specific timelines and standardize the best management protocols. Although no official recommendations exist, our center's standard of care includes patient observation twice a year and an annually orthopantomography.

Nevertheless, prevention is mandatory in this high-risk group. Stomatologists should be aware of the problems posed by HS on oral health and should provide effective and individual oral health care to such patients.
